# Case mix, outcome, and activity for admissions to UK critical care units with severe acute pancreatitis: a secondary analysis of the ICNARC Case Mix Programme Database

**DOI:** 10.1186/cc5682

**Published:** 2007-03-07

**Authors:** David A Harrison, Giovanna D'Amico, Mervyn Singer

**Affiliations:** 1 Intensive Care National Audit & Research Centre (ICNARC), Tavistock House, Tavistock Square, London WC1H 9HR, UK; 2 Department of Epidemiology and Population Health, London School of Hygiene & Tropical Medicine, Keppel Street, London WC1E 7HT, UK; 3 Bloomsbury Institute of Intensive Care Medicine, University College London, Middlesex Hospital, Mortimer Street, London W1T 3AA, UK; 4 Bloomsbury Institute of Intensive Care Medicine, 5th Floor, The Jules Thorn Building, The Middlesex Hospital, Mortimer Street, London, W1T 3AA, UK

## Abstract

**Introduction:**

Severe acute pancreatitis (SAP) requiring admission to a critical care unit is associated with high mortality and long lengths of stay. We describe the case mix, outcome, and activity of admissions with SAP who were identified from a high-quality clinical database.

**Methods:**

We conducted a secondary analysis of the ICNARC (Intensive Care National Audit & Research Centre) Case Mix Programme Database of 219,468 admissions to 159 adult, general critical care units in England, Wales, and Northern Ireland for the period of 1995 to 2003 to identify admissions with SAP. The ability of the modified Glasgow criteria to discriminate hospital survivors from non-survivors was compared to that of the Acute Physiology and Chronic Health Evaluation (APACHE) II score and a number of individual physiological parameters.

**Results:**

A total of 2,677 admissions with SAP were identified (1.2% of all admissions). Mortality for these admissions was 31% in the critical care unit and 42% in hospital. The median length of stay in the critical care unit was 3.8 days and was similar for survivors and non-survivors. Increasing numbers of modified Glasgow criteria were associated with increasing hospital mortality, but better discrimination was provided by the APACHE II score and by several physiological parameters.

**Conclusion:**

SAP requiring critical care is associated with high mortality and long length of stay. The modified Glasgow criteria represent a simple measure of severity but were not designed to predict hospital mortality. It may be possible to develop a specific model for risk prediction in patients with SAP requiring critical care.

## Introduction

Acute pancreatitis affects approximately 10 to 20 per million of the UK population per year [1] and approximately 25% of these require some form of critical care [2]. Severe acute pancreatitis (SAP) requiring admission to a critical care unit is associated with high mortality [1,3]. Management of SAP on the critical care unit is resource-intensive [4], and admissions have long stays in critical care [5]. However, data on outcomes and activity are sparse and predominantly from single specialist centres with limited numbers of patients.

A number of severity scoring approaches exist for acute pancreatitis, the most commonly used being the criteria of Ranson and colleagues [6], the Glasgow (Imrie) criteria [7], and the modified Glasgow criteria [8]. These scoring systems were devised predominantly for identifying SAP, and it is not clear how useful they are as prognostic tools in a critical care setting.

We interrogated a large national critical care database to ascertain the frequency of admissions with SAP, demographics of the patients, their first 24-hour illness severity, and subsequent outcome and activity. We applied the definitions of the modified Glasgow criteria and compared the discrimination of this score for predicting hospital mortality with that of the Acute Physiology and Chronic Health Evaluation (APACHE) II score and a number of physiological parameters.

## Materials and methods

### Case Mix Programme Database

Data were extracted for 219,468 admissions to 159 adult, general critical care units–intensive care units (ICUs) and combined intensive care/high-dependency units–from the Intensive Care National Audit & Research Centre (ICNARC) Case Mix Programme Database (CMPD) for the period of December 1995 to June 2003. Data were collected prospectively and abstracted locally by trained data collectors according to precise rules and definitions. The data underwent extensive validation before incorporation into the CMPD. The process of data validation and cleaning has been described in detail previously [9].

### Selection of cases

The primary and secondary reasons for admission to the critical care unit are coded in the CMPD by means of a hierarchical coding system, the ICNARC Coding Method [10]. Admissions were identified as having SAP if their primary reason for admission was recorded as 'acute pancreatitis,' 'chronic pancreatitis,' or 'infective pancreatitis.' Admissions transferred in from another critical care unit were excluded. APACHE II exclusions (age less than 16 years, length of stay less than 8 hours) were applied when calculating APACHE II scores only [11]. Readmissions to the critical care unit within the same hospital stay were excluded from calculation of ultimate hospital mortality and length of stay in hospital.

### Case mix

Age at admission and gender were extracted. Surgical status was defined by the source of admission to the critical care unit and (for admissions from theatre) by the National Confidential Enquiry into Patient Outcome and Death classification of surgery as elective (including scheduled) or emergency (including urgent). Three serious conditions from the past medical history were extracted using data collected as part of the chronic health evaluation section of the APACHE II score [11]. These were biopsy-proven cirrhosis documented at any time prior to or at admission to the critical care unit; hepatic encephalopathy (grade 1 or greater) occurring within the six months prior to admission; and steroid treatment, defined as more than or equal to 3 mg/kg prednisolone or equivalent daily for the six months prior to admission.

Mechanical ventilation during the first 24 hours following admission to the unit was identified by the recording of a ventilated respiratory rate. The following physiological parameters, selected *a priori*, were extracted from the data collected during the first 24 hours in the ICU: lowest arterial oxygen tension (PaO_2_), lowest PaO_2_/FIO_2_(PaO_2_/fractional inspired oxygen) gradient, lowest arterial pH, highest serum urea, highest serum creatinine, highest serum glucose, highest total serum bilirubin, lowest total serum calcium, lowest ionised serum calcium, lowest serum albumin, highest white blood cell count, and lowest platelet count. Overall acute severity of illness was summarised with the APACHE II Acute Physiology Score (APS) and the APACHE II score.

Seven of the eight modified Glasgow criteria for assessing severity of pancreatitis [8] were matched based on data available from the first 24 hours following admission to the unit. These were age older than 55 years, white blood cell count greater than 15 × 10^9^/l, serum glucose greater than 10 mmol/l, serum urea greater than 16 mmol/l, PaO_2 _less than 8 kPa, serum albumin less than 32 g/l, and total serum calcium less than 2 mmol/l. The remaining criterion that could not be matched was lactate dehydrogenase more than 600 IU/l (lactate dehydrogenase is not recorded in the CMPD). According to the original definitions, these parameters should be assessed during a 48-hour period. However, data were available for only 24 hours.

### Outcome

Mortality data were collected at discharge from the admitting critical care unit (critical care unit mortality) and at ultimate discharge from an acute hospital (ultimate hospital mortality).

### Activity

Length of stay in the critical care unit was calculated (in fraction of days) from the date/time of admission to and date/time of discharge from or death in the critical care unit. For critical care unit survivors, post-discharge length of stay in hospital was calculated (in days) from the date of discharge from the critical care unit and date of ultimate discharge from an acute hospital or death in hospital. Transfers out to another ICU were identified as critical care unit survivors whose destination following discharge from the unit was an ICU in the same or another hospital. Readmissions to the critical care unit in the same hospital stay were identified from the postcode, date of birth, and gender and were confirmed by the participating units.

### Analyses

The case mix, outcome, and activity of admissions with SAP were described using the variables defined above with numbers and percentages (for categorical variables) or medians and interquartile ranges (for continuous variables).

Ultimate hospital mortality was also reported by the number of modified Glasgow criteria and the APACHE II score. The ability of the modified Glasgow criteria to discriminate survivors from non-survivors was explored with receiver operating characteristic (ROC) curves. The ROC curve for the number of modified Glasgow criteria was compared to those for the APACHE II score and for the 12 individual physiological parameters listed above.

The effect of case mix on length of stay in the critical care unit was explored by comparing median lengths of stay by quartiles of APACHE II score and survival status. All analyses were performed using Stata 8.2 (StataCorp LP, College Station, TX, USA).

## Results

### Selection of cases

In total, 2,677 admissions were identified as SAP, representing 1.2% of all admissions in the CMPD. Of these, 2,376 (88.8%) were coded as acute pancreatitis, 159 (5.9%) as infective pancreatitis, and 142 (5.3%) as chronic pancreatitis. All were considered to represent SAP because their pancreatitis was recorded as the primary reason for admission to the critical care unit. Admissions coded with chronic pancreatitis were assumed to have a severe acute exacerbation of chronic pancreatitis.

### Case mix

The case mix of admissions with SAP is summarised in Table 1. The age and gender distributions were comparable to those of all admissions in the CMPD [9]; however, as one would expect, the majority of admissions (84%) were non-surgical. Serious conditions in the past medical history were rare, and only 8, 13, and 27 admissions met the definitions of hepatic encephalopathy, biopsy-proven cirrhosis, and steroid treatment, respectively. The median APACHE II APS (for eligible admissions) was 13, and the median APACHE II score was 17. Between 14% and 72% of admissions met each of the seven modified Glasgow criteria.

**Table 1:** Case mix for patients with severe acute pancreatitis who were admitted to adult, general critical care units

Case mix	N	Median (IQR) or *n *(%)	
Age in years^a^	2,677	63	(48–74)

Gender^b^	2,677		
Male		1,500	(56.0)
Female		1,177	(44.0)

Surgical status^c^	2,675		
Non-surgical		2,243	(83.9)
Elective/scheduled		147	(5.5)
Emergency/urgent		285	(10.7)

Past medical history^c^	2,633		
Biopsy-proven cirrhosis		13	(0.5)
Hepatic encephalopathy		8	(0.3)
Steroid treatment		27	(1.0)

Mechanical ventilation^c^	2,671	1,242	(46.5)

Physiology^a^			
Lowest PaO_2_, kPa	2,470	9.8	(8.6–11.3)
Lowest PaO_2_/FIO_2_, kPa	2,467	20.4	(13.7–29.8)
Lowest arterial pH	2,467	7.30	(7.20–7.36)
Highest serum urea, mmol/l	2,307	9.3	(5.3–16.0)
Highest serum creatinine, μmol/l	2,559	112	(78–212)
Highest serum glucose, mmol/l	2,243	8.8	(6.9–11.5)
Highest total serum bilirubin, μmol/l	1,969	20	(12–37)
Lowest total serum calcium, mmol/l	1,791	1.86	(1.62–2.08)
Lowest ionised serum calcium, mmol/l	548	1.1	(0.97–1.84)
Lowest serum albumin, g/l	2,118	22	(17–27)
Highest white blood cell count, × 10^9^/l	2,556	14.2	(10.3–19.4)
Lowest platelet count, × 10^9^/l	2,320	174	(122–240)

APACHE II^a^	2,434^b^		
Acute Physiology Score		13	(9–17)
APACHE II score		17	(13–22)

Modified Glasgow criteria^c^	2,677		
Serum albumin <32 g/l		1,926	(71.9)
Age >55 years		1,729	(64.6)
Total serum calcium <2 mmol/l		1,179	(44.0)
White blood cell count >15 × 10^9^/l		1,163	(43.4)
Serum glucose >10 mmol/l		826	(30.9)
Serum urea >16 mmol/l		573	(21.4)
PaO_2 _<8 kPa		375	(14.0)

^a^Values expressed as median (IQR). ^b^Exclusion criteria: age less than 16 years, length of stay less than 8 hours. ^c^Values expressed as number (percentage). APACHE, Acute Physiology and Chronic Health Evaluation; FIO_2_, fractional inspired oxygen; IQR, interquartile range; N, number of non-missing observations; PaO_2_, arterial oxygen tension.

### Outcome

**Figure 1:**
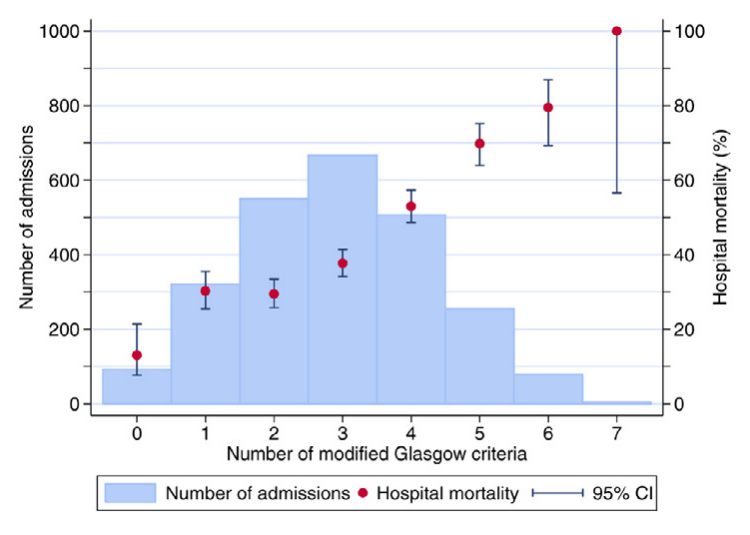
Ultimate hospital mortality by total number of modified Glasgow criteria. CI, confidence interval.

**Figure 2:**
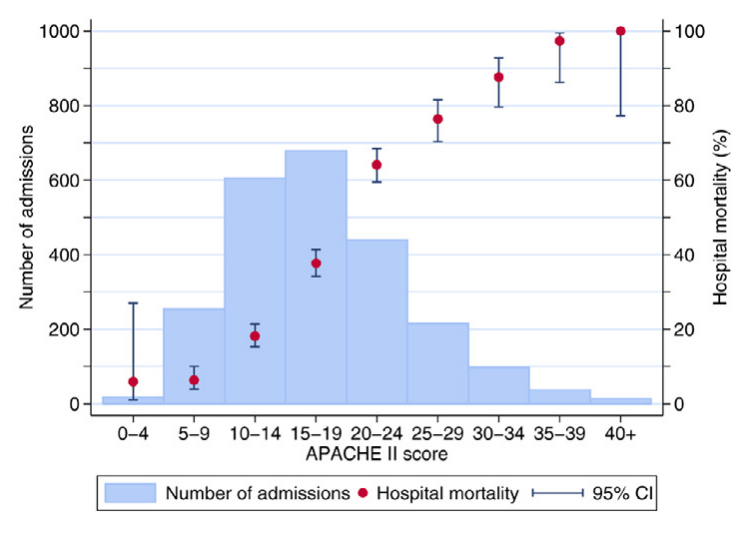
Ultimate hospital mortality by Acute Physiology and Chronic Health Evaluation (APACHE) II score. CI, confidence interval.

Of all admissions with SAP, 31% died in the admitting critical care unit (Table 2). Of all patients with SAP admitted to a critical care unit (excluding readmissions of the same patient within the same hospital stay), 42% died before ultimate discharge from an acute hospital (Table 2). Increasing numbers of modified Glasgow criteria were associated with increasing mortality, rising from 13% for admissions meeting no modified Glasgow criteria to 100% for the five admissions meeting all seven modified Glasgow criteria (Figure 1). Ultimate hospital mortality also increased with increasing APACHE II score, rising from 6% for admissions with an APACHE II score below 10 to 100% for the 13 admissions with an APACHE II score of 40 or higher (Figure 2).

**Table 2:** Unit and hospital outcome and activity for admissions with severe acute pancreatitis

Outcome		N	Deaths	Percentage
Critical care unit mortality		2,677	818	30.6
Ultimate hospital mortality^a^		2,473	1,035	41.9

Activity		N	Median	IQR

Length of stay in days				
Critical care unit	Survivors	1,854	3.7	1.6–10.1
	Non-survivors	814	3.8	1.1–13.4
	All	2,668	3.8	1.4–11.0
Post-discharge^a,b^	Hospital survivors	1,427	15	9–30
	Non-survivors	220	14	5–38
	All	1,647	15	8–30

Activity		N	*n*	Percentage

Transferred out to another intensive care unit		2,677	135	5.0

Readmission within the same hospital stay		2,677	131	4.9

^a^Excluding readmissions within the same hospital stay. ^b^Critical care unit survivors only. IQR, interquartile range; N, number of non-missing observations.

**Figure 3:**
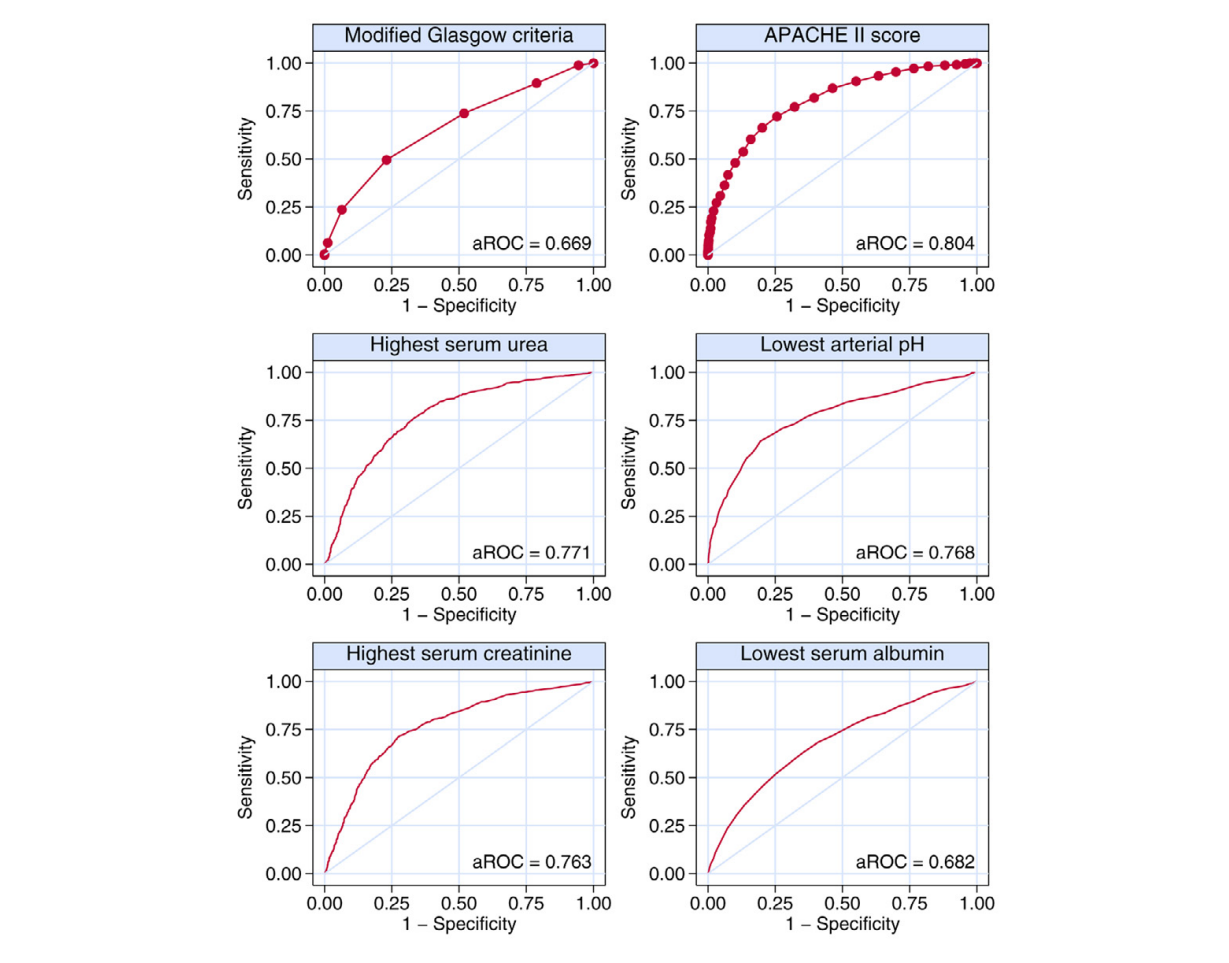
Receiver operating characteristic curves for predicting ultimate hospital mortality by number of modified Glasgow criteria, Acute Physiology and Chronic Health Evaluation (APACHE) II score, and four physiological variables. aROC, area under the receiver operating characteristic curve.

Areas under the ROC curves (aROCs) for the number of modified Glasgow criteria, APACHE II score, and 12 physiological parameters are shown in Table 3. The number of modified Glasgow criteria showed moderate ability to discriminate between hospital survivors and non-survivors (aROC = 0.669), but this was exceeded by the APACHE II score (aROC = 0.804) and four of the individual physiological parameters: highest serum urea, lowest arterial pH, highest serum creatinine, and lowest serum albumin. The ROC curves for the number of modified Glasgow criteria, the APACHE II score, and these four physiological parameters are shown in Figure 3.

**Table 3:** Area under the receiver operating characteristic curve for ultimate hospital mortality

Variable	N^a^	aROC	95% CI
Number of modified Glasgow criteria	2,473	0.669	0.648–0.691

APACHE II score	2,369	0.804	0.786–0.821

Highest serum urea, mmol/l	2,140	0.771	0.751–0.791
Lowest arterial pH	2,288	0.768	0.748–0.788
Highest serum creatinine, μmol/l	2,370	0.763	0.744–0.783
Lowest serum albumin, g/l	1,972	0.682	0.658–0.706
Lowest PaO_2_/FIO_2_, kPa	2,291	0.660	0.638–0.683
Lowest total serum calcium, mmol/l	1,663	0.617	0.589–0.645
Lowest platelet count, × 10^9^/l	2,152	0.599	0.574–0.623
Lowest PaO_2_, kPa	2,291	0.565	0.542–0.589
Highest serum glucose, mmol/l	2,078	0.560	0.534–0.585
Highest total serum bilirubin, μmol/l	1,826	0.545	0.518–0.572
Lowest ionised serum calcium, mmol/l	515	0.527	0.476–0.578
Highest white blood cell count, × 10^9^/l	2,368	0.524	0.500–0.549

^a^Excluding readmissions within the same hospital stay and admissions missing ultimate hospital outcome. APACHE, Acute Physiology and Chronic Health Evaluation; aROC, area under the receiver operating characteristic curve; CI, confidence interval; FIO_2_, fractional inspired oxygen; N, number of non-missing observations; PaO_2_, arterial oxygen tension.

### Activity

**Figure 4:**
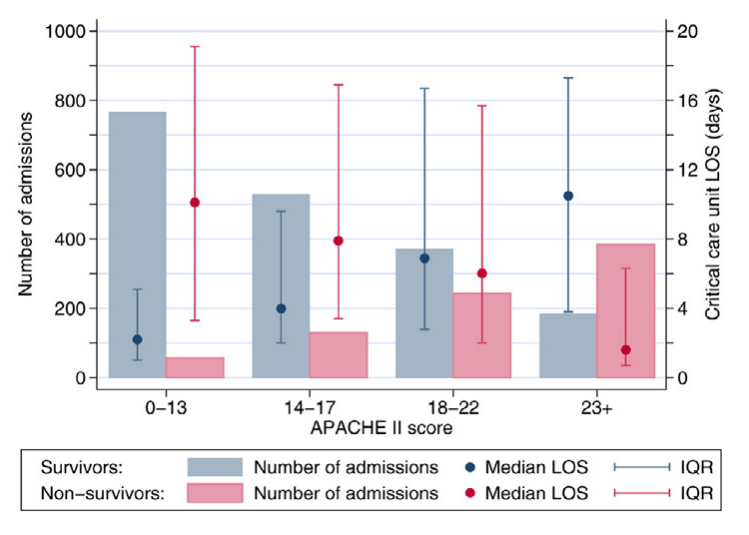
Length of stay (LOS) in the critical care unit by Acute Physiology and Chronic Health Evaluation (APACHE) II score and survival status. IQR, interquartile range.

Overall median length of stay in the critical care unit was similar for survivors and non-survivors (3.7 versus 3.8 days) (Table 2). However, median length of stay increased with increasing severity of illness for survivors and decreased with increasing severity of illness for non-survivors (Figure 4). Length of stay in hospital after discharge from the critical care unit was also similar for hospital survivors and non-survivors (median 15 versus 14 days) (Table 2), although the lengths of stay of non-survivors were more variable (interquartile range 5 to 38 days compared to 9 to 30 days for survivors). Five percent of all admissions with SAP were transferred out to another ICU, and 5% of admissions represented a readmission of a patient previously admitted during the same hospital stay (Table 2).

## Discussion

This paper presents case mix, outcome, and activity data for a large cohort of patients with SAP who were identified from a high-quality clinical database. The modified Glasgow criteria were found to provide a simple score that summarised the severity of acute pancreatitis, with an increasing number of modified Glasgow criteria associated with increasing mortality and providing moderate discrimination between hospital survivors and non-survivors. However, better discrimination was provided by the APACHE II score and by several individual physiological measurements.

The main strength of this study is the large cohort, which is representative of all patients with SAP who were admitted to adult, general critical care units in the UK. Previous studies of a comparable size have been based on hospital episode statistics [12-15], which do not allow the analysis of detailed physiological measurements which was possible in this study. Previous studies with detailed clinical data have been much smaller and have tended to concentrate on admissions from single or small numbers of specialist centres. Even the studies used to develop scoring systems have been based on between 100 [6] and 405 [8] patients with pancreatitis.

The main weakness of this study is the reliance on data collected for a different purpose. Of the severity scoring systems for acute pancreatitis, we opted to use the modified Glasgow criteria as these had the greatest overlap with the data included in the CMPD. Even so, data were not available for one of the eight criteria (lactate dehydrogenase), and it was necessary to score the criteria during a 24-hour period rather than the prescribed 48 hours. This may reduce the utility of the modified Glasgow criteria for predicting outcome as some studies have noted that severity of pancreatitis may worsen after the first 24 hours [16,17]. Furthermore, the modified Glasgow criteria were not designed for predicting outcomes in patients with SAP, but rather to identify SAP.

Consensus criteria exist for the definition of SAP [18], but it was not possible to match these criteria in the CMPD. We feel that a diagnosis of pancreatitis as the primary reason for admission to a critical care unit can be considered to be a reasonable pragmatic definition of SAP. The lead author of the consensus criteria has also noted that in the more than 10 years since their conception, they remain poorly validated [19].

A consensus conference on the management of critically ill patients with SAP in 2004 [20] led to the first international recommendations regarding the question of when patients should be admitted to a critical care unit. The consensus group concluded that the use of disease-specific and global severity scores may assist in the identification of high-risk patients but should not replace close clinical observation. The levels of discrimination observed in this study support this recommendation.

## Conclusion

SAP requiring admission to a critical care unit is associated with high mortality and long length of stay; this review identified 2,677 admissions to 159 critical care units with hospital mortality of 42% and a median length of stay in critical care of four days. Although the modified Glasgow criteria provide a simple tool for summarising the severity of acute pancreatitis, they were not designed for predicting hospital mortality in patients with SAP requiring critical care. Good discrimination between survivors and non-survivors was obtained by using the APACHE II score. Good discrimination was also found for four individual physiological parameters–highest serum urea, lowest arterial pH, highest serum creatinine, and lowest serum albumin–suggesting that it may be possible to produce a new score to provide a more accurate outcome prediction for this population.

## Key messages

• SAP requiring admission to a critical care unit is associated with high mortality and long length of stay.

• Increasing numbers of modified Glasgow criteria were associated with increasing hospital mortality, although the criteria were not designed for this purpose and only seven of the eight criteria could be identified in these data.

• Good discrimination was obtained with the APACHE II score and with four individual physiological parameters: highest serum urea, lowest arterial pH, highest serum creatinine, and lowest serum albumin.

## Abbreviations

APACHE = Acute Physiology and Chronic Health Evaluation; APS = Acute Physiology Score; aROC = area under the receiver operating characteristic curve; CMPD = Case Mix Programme Database; FIO_2 _= fractional inspired oxygen; ICNARC = Intensive Care National Audit & Research Centre; ICU = intensive care unit; PaO_2 _= arterial oxygen tension; ROC = receiver operating characteristic; SAP = severe acute pancreatitis.

## Competing interests

The authors declare that they have no competing interests.

## Authors' contributions

MS conceived the study. DAH performed the analyses and drafted the manuscript. GD'A performed the referencing. All authors participated in the interpretation of the study and critical revision of the manuscript and have read and approved the final manuscript.
